# Sugar Addiction: From Evolution to Revolution

**DOI:** 10.3389/fpsyt.2018.00545

**Published:** 2018-11-07

**Authors:** David A. Wiss, Nicole Avena, Pedro Rada

**Affiliations:** ^1^Fielding School of Public Health, University of California, Los Angeles, Los Angeles, CA, United States; ^2^Icahn School of Medicine at Mount Sinai, New York, NY, United States; ^3^School of Medicine, University of Los Andes, Mérida, Venezuela

**Keywords:** obesity, food addiction, drug addiction, sucrose, feeding behavior, dopamine, acetylcholine, nucleus accumbens

## Abstract

The obesity epidemic has been widely publicized in the media worldwide. Investigators at all levels have been looking for factors that have contributed to the development of this epidemic. Two major theories have been proposed: (1) sedentary lifestyle and (2) variety and ease of inexpensive palatable foods. In the present review, we analyze how nutrients like sugar that are often used to make foods more appealing could also lead to habituation and even in some cases addiction thereby uniquely contributing to the obesity epidemic. We review the evolutionary aspects of feeding and how they have shaped the human brain to function in “survival mode” signaling to “eat as much as you can while you can.” This leads to our present understanding of how the dopaminergic system is involved in reward and its functions in hedonistic rewards, like eating of highly palatable foods, and drug addiction. We also review how other neurotransmitters, like acetylcholine, interact in the satiation processes to counteract the dopamine system. Lastly, we analyze the important question of whether there is sufficient empirical evidence of sugar addiction, discussed within the broader context of food addiction.

## Introduction

Obesity has become one of the biggest health care burdens since the second World War ended, increasing morbidity and lowering life expectancy (1, 2). It is a major contributing factor to several chronic conditions, including cardiovascular disease, diabetes, and cancer (3). Given the social and economic burden associated with the “obesity epidemic” there has been considerable global interest across many disciplines including medicine, nutrition, neuroscience, psychology, sociology, and public health in order to reverse this trend. Numerous interventions have been proposed, but there has been minimal progress to date. This obesity crisis affects not only developed countries but less developed ones as well, with up to 30% or more of its population categorized as overweight or obese (1, 4). The disproportionate increase in body weight has intensified in the last 30 years (1, 5, 6).

Virtually all investigators have asked the question of what has changed in this relatively short period of time? A common theory is an increase in sedentary lifestyles. Some contend that this alone explains the epidemic, arguing that energy expenditure, rather than food consumption, has significantly decreased in modern society compared to our hunter-gatherer ancestors (7). Multiple studies support this concept of a direct correlation between physical inactivity, television watching hours, and obesity (8–10). A second theory is the availability and consumption of highly palatable foods, which has surged in the past few decades. Nestle reported the appearance of 11,000 new food products added to the supermarket shelves every year in 1998 (11), introducing countless new and attractive flavor combinations for food consumers. Investigations into the link between the “food environment” and obesity have led to the conclusion that ubiquitous access to relatively inexpensive and convenient “snack” foods have changed normal eating behavior, including less time spent preparing meals at home (12). Industrialization of the food supply has decreased the cost of energy dense foods by adding refined sugars, grains, and/or fats to their products (11). Consumption of these processed foods has increased in children (13) and toddlers (14).

While behavioral and lifestyle interventions remain the mainstream “treatment” approach for obesity, dietary adherence remains an obstacle (15). Recent research suggests that highly processed foods are addictive and the hedonic mechanisms (pleasure-seeking pathways) may play a critical role in the pathogenesis of obesity (16). It has also been suggested that the focus on calorie counting is misguided, and that future strategies should emphasize dietary quality and individual factors such as hormonal regulation of metabolism (17), and the gut microbiome (18). Given the challenges that many people face controlling their appetites in today's “food environment” it appears that public policy changes will be required to modify the conditions in which food choices are made (19). According to Gearhardt and Brownell (20) “it will be important to look at the widespread subclinical impact of potentially addictive foods through the use of public health approaches” (20). The goal of this paper is to review the human predilection for refined sugars and how they reshape the brain, with its implications for public health policy.

## The nutrition transition theory

The nutrition transition theory first emerged to describe global trends toward a “Western diet” containing refined foods high in fat and sugar, and low in fiber (21). Later the term was used to capture a correlation with increased BMI and changing economic and agricultural factors. Early identified factors include urbanization, economic growth, technical change, and culture (22) while more recent descriptions of the critical underlying factors include technology, urbanization, economic welfare relative to the cost of food, and expansion of global trade (23). The nutrition transition theory is not a new concept. Previous models have included the demographic and epidemiological transitions. Popkin and Gordon-Larsen identify that both historic processes precede the nutrition transition (22). The epidemiological transition describes a shift from high prevalence of disease associated with famine, malnutrition, and poor sanitation, to a pattern of high prevalence of chronic and degenerative disease associated with urban-industrial lifestyles (24). This ecological framework analyzes changes at the societal level, examining how agricultural and food supply chains impact global dietary patterns. The theory suggests that “upstream” interventions (supply-side) will be more effective than addressing the lower hanging fruit (i.e., exercise, calorie restriction).

The nutrition transition theory is also supported by compelling evidence suggesting that a wide range of animals have also been gaining weight in recent years (25, 26). Other terms that support the “environmental theory of obesity” include “globesity” at the most distal levels, and the “neighborhood effect” at more proximal levels (27). Notwithstanding, the “neighborhood effect” has far-reaching social implications, given that the neighborhood where one lives is merely a proxy for socioeconomic status. Recently, other research has suggested that discussions of nutritional inequality emphasizing supply-side factors are less indicative of consumption patterns than demand-side differences (28), lending support to the food addiction (FA) hypothesis.

## Evolutionary and genetic aspects of feeding

Adipose tissue in mammals play an important role in survival by preparing the body for periods of famine (29). From an evolutionary perspective, the increase in body fat prepared animals for times of food scarcity, in fact, those accumulating body fat had an advantage compared to those that did not (30). However, this occurred in times when humans had insecure food supply (hunter-gatherer) and could spend many days on a hypocaloric diet. During prehistoric times, the excessive increase in body weight was dampened by physical activity needed in the search of food, moreover, excessive fat would mean, as a predator, lower chances of catching the prey and vice versa (29). So, even if copious quantities of food were eaten, there was a natural brake mediated by physical activity.

When did this panorama change? The first change was the advent of agriculture and animal domestication ~10,000 years ago, leading people to become producers by gathering and securing food supply (31). Of course, farming depended on climate and plagues which could decimate crops resulting in famine (3). The second change was the industrialization of food supply (industrial revolution of the nineteen century) allowing for mass production of flour and sugar (32), with the ulterior manufacturing, in the last decades, of processed and ultra-processed foods that are inexpensive and highly caloric (abundant sugars, salts, fats) (11, 33). These two developments are linked to food availability and how food is refined and commercialized. Meanwhile, a third important revolution happened over the past few decades: the arrival and public accessibility of automobiles, television sets, and later the computer leading us toward a sedentary lifestyle (7). When all three transformations are combined, we can see that caloric intake has risen while calorie expenditure has significantly decreased, leading to the obesity epidemic.

Although humans have culturally and technologically evolved, our genome has changed very little in the last 10,000 years (4). This means that our brain circuitry is still programmed to eat more in times of food abundance preparing for periods of starvation (31). Recent genetic studies have focused on gene polymorphisms related to specific nutrients and obesity (34–37). This area of investigation has been called nutrigenetics and suggests that epigenetic factors influence the expression of predisposing genes in certain populations. For example, positive associations have been found between the fat-mass and obesity associated gene (FTO) and BMI (38). Many investigators are interested in genes such as beta-adrenergic receptor 2 (ADRB2) and melanocortin receptor 4 (MCR4), since their expression may be altered following ingestions of carbohydrates (sugar) (39–41). Researchers have found a significant interaction between sugar-sweetened beverages and genetic-predisposition score calculated on the basis of 32 BMI-associated loci suggesting that people carrying this trait, when exposed to sweetened beverages, BMI, and adiposity will be augmented (35). In addition, other investigators have found that at chromosome16p11.2 different variations of this gene can affect consumption of sweet foods (42, 43). The question at this point is: how can we link sugar ingestion to addictive behavior?

## Evolution of addictive drugs

When Charles Darwin postulated the evolution theory, he suggested that a trait would emerge if it contributed to survival and increase the reproductive success of a species. Plants have evolved protective measures to prevent herbivores from eating them. For example, some alkaloids that give the plant a bitter taste cause avoidance by most species in the animal kingdom (44, 45). Nonetheless, many animal species including hominids, as well as prehistoric humans, ingested lesser amounts of toxic substances and obtained benefits for their own survival (45). Thus, a coevolution occurred as different traits were evolving in animals for the detection of caloric nutrients in foods (i.e., carbohydrates), traits emerged that permitted the ingestion of small amount of toxic plants to prevent diseases or to improve physical conditions (45). This would explain the chewing of cocaine or tobacco leaves by aborigines in the Americas allowing them better physical fitness to cope with fatigue and a better chance to catch prey or find food (44). One could argue that, like our dependency on nutritive foods to survive, we were also partially dependent on certain toxic plants. What made them addictive? Analogous to nutrients, humans learned how to process these toxic plants, increasing their potency, as it is done in modern times, conferring drugs and foods with a salient rewarding response. Thus, in both cases (food or drugs) an “evolutionary mismatch” has occurred by which human technology has been able to alter environmental conditions much faster than the changes that occur in our central nervous system (46, 47). Ultimately, early in our evolution the ingestion of food or drugs emerged as positive reinforcement and evolved common neural circuits for reward, and that has not changed over time, owing to their sharing of similar neural mechanisms in addictive behavior (48–50).

## Neural circuits for reward

The limbic system consists of different brain regions engaged in various aspects of emotions. Historically, it included a bidirectional pathway between the hippocampus and the hypothalamus (51). Over time, other structures have been added to the circuit including: the amygdala, the nucleus accumbens (ventral striatum) and the prefrontal cortex. The functions of these structures are complex, and their diverse mechanisms of action are still being elucidated. Various neurotransmitters in this circuit (like GABA, glutamate, and opioids) are involved in several aspects of reward (52, 53), however, the dopaminergic pathway from the ventral tegmental area (VTA) to the nucleus accumbens (NAc) has received the most attention in the “reward” cascade (54–60). To summarize, blocking the dopaminergic pathway between the VTA and the NAc inhibits instrumental responding for food and became the foundation of the dopamine (DA) hypothesis of reward (61). Later, studies have demonstrated that “reward” is a vague term (62) that consists of at least three components: hedonics (“liking”), reinforcement (learning) and motivation (incentive, “wanting”) (63). DA in the NAc seems to have a preponderant role in the latter two components (learning and incentive motivation) and less in the former (hedonics) where the opioid and GABA system appear to play a stronger role (64, 65).

## Food “reward” and accumbens dopamine

Although the exact contribution of accumbens DA in reward is still unclear, most researchers agree that it is involved in feeding behavior. For instance, original studies in the 1970's have shown that a lesion in the striatonigral DA pathway with 6-OH-dopamine provoked a profound aphagia and adipsia (66). This finding was later corroborated in DA-deficient mice that also became hypoactive, aphagic, and adipsic (67). Similarly, lever pressing for food pellets in animals increases DA release in the NAc (68–70), however, not during rat chow free-feeding (70, 71) suggesting that DA in the accumbens regulates instrumental learning. Others have observed that accumbens DA increases during rat chow feeding only if rats were food deprived (72, 73) or in the presence of palatable foods (74–81). Interestingly, increased DA while eating highly palatable food wanes after repeated exposure (74, 75, 79) and this returns if palatable foods are switched to a different one (82) suggesting a role of this neurotransmitter in the NAc for novelty recognition. Additionally, it has been shown that DA neurons respond to exposure of a novel food and if that novel food is paired with a cue, in a subsequent exposure, food alone will not induce neuronal firing while the cue alone does, suggesting that DA neurons are involved in conditioned learning (83, 84). Cue-invigorating food-seeking may be considered adaptive, but the maladaptive eating in the absence of hunger forms the basis for the FA hypothesis. It has been shown that limited or intermittent access to highly palatable foods increase cue-reactivity to these foods, which has implications for the consequences of extreme dieting behavior in humans (85).

Another preponderance of evidence for the engagement of accumbens DA on feeding behavior comes from studies utilizing orexigenic peptides. It is well known that some peptides in various brain sites are capable of initiating feeding behavior, for instance, paraventricular injection of galanin, ghrelin, or opioids will promote food intake even if rats are satiated (86–92). These peptides, systemically or locally injected in the paraventricular nuclei, increased NAc DA (93–95). Inversely, local injection of cholecystokinin (CCK), an anorexigenic peptide, decreased DA release in the NAc (96). It appears that accumbens DA plays more of a role in the anticipatory behavior than in the consummatory behavior. Stomach-derived ghrelin has known action on orexigenic neurons in the hypothalamus, and receptors have been identified in the VTA, hippocampus, and amygdala (97, 98). Ghrelin appears to be implicated in rewarding aspects of eating distinct from homeostatic mechanisms that promote food consumption when energy stores are low, thus may be a key driver in the motivational aspects (“wanting”) of consuming palatable foods beyond metabolic need (99, 100).

Finally, pharmacological manipulation of the DA system has led to contradictory results. On the one hand, DA injected directly into the NAc is capable of increasing ingestive behavior (101, 102). However, others have not been able to modify feeding behavior when specific DA agonists or antagonists were used (103, 104). Recently, chemogenetically activating DA neurons in the VTA that project to the NAc disrupted feeding patterns (105). In part, these dissimilar findings show that it is very hard to propose that only one neurotransmitter or hormone is responsible for driving behavior.

## Dysfunction of the dopaminergic system in obese subjects

Investigators can identify animals that have propensity to become obese in a 5-day weight gain on a high-fat diet (OP rats) (106). In these OP rats, a deficit of exocytosis mechanisms in the DA neuron was found, as well as a decrease in accumbal DA basal levels (107, 108). Similarly, rats made obese with a “cafeteria diet” exhibited decreased basal levels of DA in the NAc, and show a blunted DA response to the taste of rat chow, while increasing DA release in response to a highly-palatable food (109). Human studies using neuroimaging determined that obese patients had a lower sensitivity of the accumbens DA (110) and a decrease in DA-D2 receptor availability (111, 112). Several studies have used the term “reward deficiency syndrome” to describe a genetic dysfunction of the DA-D2 receptor leading to substance-seeking (food, drugs) behavior in humans (113–115). Variations in the DA-D2 gene have also been associated with impulsivity and a preference for smaller more immediate rewards compared to larger but delayed ones (delay discounting) (116). It is possible that obese subjects compensate for the depressed DA basal levels by overeating palatable foods (57). Conversely, optogenetical-induced increase in basal DA release inhibits consumption behavior (117). How can these results be reconciled with other studies? DA is released phasically and tonically with possible divergent tasks (118, 119). Basal DA levels are likely to determine the tonic response of the system, thus could confer a complete opposite response.

## Drugs of addiction and accumbens dopamine

Most drugs of addiction activate the VTA-NAc pathway whether they are systemically injected (120) or locally applied in the accumbens (121, 122). Furthermore, drugs that increase DA release in the NAc are also self-administered (123–126). Thus, drugs of addiction, like food, increase DA release in the NAc, however with drugs, this increment occurs repeatedly every time it is given, compared to a decline in release observed with palatable food. Blunted striatal DA and decreased DA-D2 receptor availability (measured using radiotracers as binding potential relative to nonspecific binding) have been repeatedly identified in position emission tomography (PET) scans of drug-addicted human subjects and is likely to be both a result and a cause of an addictive disorder (127). Given the similarities in human PET scans between drug abusers and obese subjects (128), additional research is needed to identify neurobiological risk factors for addiction-like eating. Animal studies suggest that overconsumption of each can be a predisposing factor for the other (129, 130).

## Accumbens acetylcholine and satiety signaling

Acetylcholine (ACh) is released by local interneurons that compromise less than 2% of neurons in the NAc (131, 132). They have an extensive axonal arborization and form synapses in the medium spiny output neuron (133). The idea that ACh opposes DA function in the striatum comes from research on Parkinson's disease (PD). It is known that anticholinergic (antimuscarinic) drugs were the first medications used in the treatment of PD antagonizing mainly M1 receptors (134, 135). This indicates that DA normally exerts an inhibitory action on striatal ACh interneurons as demonstrated in rats (136). In addition, L-dopa induced hyperlocomotion in DA-deficient mice is suppressed by cholinergic agonists (137). Separately, anticholinergic drugs are abused (138) probably by increasing DA activity in the striatum (139), thus, an antagonistic association probably exists between DA and ACh in the NAc and striatum.

ACh in the NAc appears to have a modulatory effect on feeding behavior. During free-feeding, ACh increased at the end of a meal (72) and during ingestion of a palatable food it reached a maximum after the animal stopped eating (79, 140). This increment disappeared in sham-fed animals that had a gastric fistula opened compared to controls with a closed gastric fistula (141). Bilateral perfusion in the NAc of the indirect ACh agonist, neostigmine, reduced food intake in food-deprived animals (142). Conversely, lesion of the cholinergic interneuron in the NAc with a specific toxin (AF64A) produced a significant increase in food intake (142). Moreover, injection of the anorectic drug combination phentermine/fenfluramine increased ACh release in the NAc (143). All these results suggest that ACh in the NAc probably signals satiety. Recently, researchers found that increasing the activity of the cholinergic interneuron in the NAc reduced palatable food consumption, lending support to the hypothesis that NAc-ACh acts as a stop signal (144).

What happens if food becomes an aversive stimulus? Using a conditioned taste aversion paradigm, it has been shown that the aversive stimulus (in this case saccharin) would decrease DA release (78) while increasing ACh output (145). Furthermore, injection of neostigmine (indirect ACh agonist) is sufficient to provoke a conditioned taste aversion (146). Therefore, an increase in DA simultaneous to an increase in ACh release in the NAc signals satiety (stop) but if the change in release of these neurotransmitters is divergent (decrease in DA and increase in ACh) then the stimulus becomes aversive (140). Taken together, animal feeding induces an initial and long-lasting increase in DA release followed by an increase in ACh output signaling satiation, making the animal feel satisfied (DA release) and stop the behavior (ACh).

## Effect of drugs of abuse and withdrawal on acetylcholine release in the NAc

Drugs of addiction exert differential responses on the accumbens cholinergic interneuron. One could separate these drugs by their effect on feeding, for example, ACh release is decreased or not changed in the NAc if the drug increases food intake (opioids, alcohol, benzodiazepines) (147–150) while those that act as anorectic (cocaine, amphetamine, nicotine) produce the opposite effect, an increase in ACh release (142, 151–153). Moreover, cholinergic ablation in the NAc increased sensitivity to cocaine (154). What is common for most drugs of addiction is that during drug withdrawal ACh is increased in the NAc (147, 149–151, 155). In addition, enhanced functioning of the ACh interneuron in the NAc prevents addictive behaviors for cocaine and morphine (156). The augmented release of ACh in the NAc occurs simultaneously to a decrease in DA release (150, 151, 155, 157), identical to the response observed during a conditioned taste aversion.

## What is the difference between food and drugs of addiction?

First, feeding behavior, as with other “natural” behaviors, has a satiety system provided by the mechanical limitations of the stomach and peptides like CCK that signal satiety while drugs of addiction apparently do not. Secondly, even in the presence of a palatable meal, the pleasant effect seems to wane simultaneous to a blunting of the DA response (74, 75, 79, 82) even though in some cases “sensory-specific satiety” can lead to continued consumption behavior after a novel food is introduced (82). Finally, the magnitude of the DA increase is lower during meal than during drug administration. Drugs of abuse not only release striatal DA but also block or reverse DA reuptake, creating a more potent reinforcement through the euphoric state (158). Some authors have made the argument that there is no concrete evidence of withdrawal from food, especially when compared to drugs like opioids (159) and that calling food addictive risks trivializing more serious addictions (160). Other arguments against FA have suggested “eating addiction” as behavioral rather than substance-related (161). Evidence of withdrawal in animal models will be reviewed below.

Given that adolescence is a critical period of neurodevelopment, it appears as though exposure to sucrose during this time (rodents from postnatal day 30–46) leads to an escalated intake during the exposure period and a subsequent decrease in c-Fos-immunoreactive cells in the NAc (measured at postnatal day 70) which is involved in the processing of hedonic properties of sweet foods (162). In this experiment, adult rats consumed less sugar after heightened exposure in the adolescent period, which is consistent with other findings (163, 164). These studies also demonstrate that sugar-exposed adolescents exhibit higher preference for cocaine (164) but not alcohol (163) in adulthood. Differences in the neurobiological substrates underlying intake behavior of food and drugs abuse are likely explained by changes in the motivational aspect of food intake rather than by deficits in hedonic processing (162). These findings point to deficits in the “liking” component of sweet foods and drinks which offers insight into our understanding of reward-related disorders. Interaction effects between genetic predisposition to addiction and exposure to sugar during adolescence on the “wanting” mechanism in adulthood warrants further study.

## Can sugar be addictive?

Before we can make a case for sugar as an addictive substance, we must first define addiction, which is now referred to as substance use disorder (SUD). The American Psychiatric Association defines addiction, in its web page for patients and family, as “a complex condition, a brain disease that is manifested by compulsive substance use despite harmful consequence.” Operationally, experts utilize the Diagnostic and Statistical Manual of Mental Disorders (DSM) as a tool to unify diagnostic criteria in clinical and/or experimental design. The current version of this manual known as the DSM-5 includes a section for SUD and it incorporates eleven criteria for diagnosis. A patient must fulfill at least two of these criteria. In turn, these eleven criteria, by their characteristics, can be compiled into four broader groups (165) (see Table 1).

**Table 1:** Four broader categories for eleven criteria used for substance use disorder (SUD).

A. Impaired Control	1. Use larger amount and for longer than intended. 2. Craving. 3. Much time spent using. 4. Repeated attempts to quit and/or control use.
B. Social Impairment	1. Social/interpersonal problems related to use. 2. Neglected major role to use. 3. Activities given up to use.
C. Continued use Despite Risk	1. Hazardous use. 2. Physical/psychological problems related to use.
D. Pharmacologial Criteria	1. Tolerance. 2. Withdrawal.

These guidelines are designed to help with the diagnosis of patients, however, scientists use them in animal models, discarding those that are unique to human behavior (i.e., social impairment). Our animal model for sugar addiction consists of rodents with restricted access to 10% sugar or 25% glucose solution during a 12-h period starting 4 h into their active cycle (as Bart Hoebel would remark “animals skipped breakfast”) for 21 days (details of the protocol can be found in Avena et al. (166). We are able to examine the following criteria met by our model:

- Impaired control:

1. Use larger amounts and for longer than intended: rats will typically escalate their sugar ingestion progressively from an initial 37 mL up to 112 mL by day 11 when they reach an asymptote that persists for the next 10 days (79, 167). Escalation cannot be attributed to neophobia which is easier to overcome. In addition, experimental and control animals drink about 6 mL in the first hour during the first day and doubles in experimental subjects (over 12 mL) on day 21, while controls (ad lib sugar) drank the same 6 mL as the first day (79, 167). This increase could be considered a “binge” (168). Certainly, the gastrointestinal system has intrinsic mechanical restraints limiting the amount consumed during escalation of a sugar solution, if bypassed (i.e., with a gastric fistula), rats will binge over 40 mL in the first hour (141). So, intermittent administration of sugar mimics those used for drug self-administration (169) and creates a “binge” pattern of intake that resembles the compulsive behavior seen in drug abuse (170, 171). Binge-like consumption patterns of sucrose has been associated with decreased dendritic length of the NAc shell which supports the formation of increased excitatory inputs (172). Ghrelin's ability to interact directly with DA reward circuitry and ACh receptor gene expression in the VTA has been implicated in the motivational aspects of feeding under high-sugar conditions (173) which is consistent with findings that ghrelin is necessary for reward from alcohol (174, 175) and drugs of abuse (176). Meanwhile, a shortcoming here is that we cannot determine “intention” in our animal model the way it can be assessed in humans. Therefore, “intended” is an assumption.
2. Craving: defined by the Cambridge Dictionary as “a strong feeling of wanting something” or “feeling of desire.” In laboratory conditions, it is defined as the motivation (“wanting”) to obtain an abused substance (177) and is indirectly studied in animal models using instrumental behavior. In one case, rats bar press to self-administer drugs of abuse and when forced to abstain they will keep pressing the bar although unrewarded (resistance to extinction). Second, rats will readily press the bar in presence of a cue that was previously associated to the drug (incubation) (178–182). A third paradigm, used initially in alcohol addiction, is the alcohol-deprivation effect (ADE). Alcohol drinking rats will increase their consumption following an abstinence period (181, 183). Experiments carried out in rats trained to respond for sucrose, instead of drugs of abuse, exhibited resistance to extinction and incubation much like cocaine (184). Moreover, the incubation response was attenuated by naloxone administration arguing in favor of the endogenous opioid involvement in sugar craving (185). Additionally, rats trained to drink a non-caloric solution (saccharin) also showed incubation, consequently, the phenomenon depends on taste (hedonic) and not just the caloric content of the solution (186). Lastly, rats trained for 28 days to drink a sucrose solution and deprived for 14 days displayed a sugar-deprivation effect analogous to ADE (187). These results are an indirect measure of the motivation to use sugar (craving) and fulfills one of the DSM-5 criteria for SUD. Craving has been intimately related to high rates of relapse in drugs of abuse (171) and now with sugar.

B. Social impairment (unable to assess with animal model).
C. Continued use despite risk:

1. Hazardous use: In the context of drug abuse, a conditioned suppression paradigm is utilized as an indicator of a compulsive behavior and gives indirect evidence of the power of craving (168). Animals will seek a drug (i.e., cocaine) despite an aversive conditioned stimulus (188). The results on sucrose consumption, using this paradigm, is controversial. On one hand, it was found that the conditioned stimulus suppressed sugar intake indicating that the animal would not take the risk (188). In this case, rats were trained to obtain sucrose on a “seeking/taking” chain schedule that paralleled cocaine use, and the conditioned stimulus suppressed sucrose intake as well as increased seeking latency, however, in this paradigm we do not know whether rats were sugar-dependent or not. Meanwhile, others have found that mice on a highly palatable food diet were insensitive to the aversive conditioned stimulus (189–191) or would withstand an unpleasant environment to gain access to the meal (192). Further research is needed to determine if sugar dependent rats will endure an aversive stimulus to seek the sugar solution.

D. Pharmacological criteria:

1. Tolerance: is the gradual decrease in responsiveness to a drug demanding an increase in doses consumed to obtain the same initial effect (168, 193). In our model, rats progressively escalated their sugar intake as explained above and it probably argues in favor of a tolerance effect (79, 167).
2. Withdrawal: corresponds to a set of signs and symptoms that a drug user presents once the drug is suspended or the specific antagonist is injected. One of the most clearly defined, in animals, are the signs of opiate withdrawal either spontaneous or induced with a specific antagonist (i.e., naltrexone, naloxone) including: wet-dog shakes, teeth-chattering, piloerection, diarrhea, grooming, rearing, writhing (149). Two other symptoms in opiate withdrawal are anxiety and behavioral depression. The former is inferred in rats using the plus-maze and measuring the amount of time spent in the open or closed arms (194). Spontaneous and naloxone-induced opiate withdrawal in rats decreased exploration into the open arms confirming the anxiogenic-like effect following the abandonment of the drug (195). The latter symptom is explored using the forced-swim test and monitoring the amount of time swimming (196). Morphine withdrawal causes a prolonged enhancement of immobility in rats confirming the behavioral depression induced when the drug was suspended (197).

Sugar acts as an analgesic most likely by releasing endogenous opioids (198). Hence, it is sensible to look for signs of opiate withdrawal in rats made dependent on sugar or palatable food (199). Injection of naloxone in sugar-dependent rats generated several of the opiate withdrawal symptoms and anxiety-like response on the plus-maze (200, 201). Similarly, sugar deprivation (analogous to spontaneous drug withdrawal) produced signs of opiate withdrawal including anxiety-like behaviors (200, 202). Only recently have withdrawal symptoms been elucidated in humans meeting criteria for FA by way of predictive reference resetting (allostasis) controlled by the rostral anterior cingulate cortex and the dorsal lateral prefrontal cortex (203).

Neurochemically, morphine withdrawal is accompanied by a decrease in accumbens DA release with a simultaneous ACh increase (147, 149, 157). An equal response was observed when sugar experienced rats were injected naloxone or sugar deprived (200–202), confirming the involvement of the endogenous opioid system in the development of sugar dependency.

## Additional aspects of sugar addiction are comparable to drug addiction

So far, this model of sugar addiction meets five of the criteria established in the DSM-5. In addition to the clinical criteria, there are other behavioral and neurochemical attributes observed in animal experimentation that we will discuss below.

Behavioral sensitization is a phenomenon linked to several facets of drug dependence and consists on a long-lasting increase in locomotor activity following repeated administration of psychostimulants or opioids (204–206). Animals sensitized with one drug of abuse often show the same hyperactivity when a different drug is injected. This has been called cross-sensitization and occurs between different drugs of addiction (207). For example, rats sensitized to 9-delta-tetracannabinol displayed a sensitized behavior when morphine was injected (208). Equally, rats sensitized to cocaine are cross-sensitized to ethanol and vice-versa (209). Comparable to drugs of abuse, sugar dependent rats show cross-sensitization to drugs of abuse and the other way around. For instance, rats maintained on an intermittent sugar schedule display cross-sensitization to amphetamine (210) and rats sensitized to amphetamine increase their locomotion when exposed to 10% sucrose solution (211). Furthermore, sucrose intake has been shown to enhance behavioral sensitization induced by cocaine and ethanol (212, 213). Thus, intermittent sugar promotes behaviors observed with drugs of abuse.

Human research on behavioral sensitization has been used to explain the progressive nature of drug use and the role of internal and external cueing in the motivation process. Highly caloric food elicit the strongest DA response but it has been suggested that only a subset of susceptible individuals become conditioned for behavioral sensitization (214) likely due to genetic variability in the dopaminergic system. There is still some debate if individuals are more susceptible under conditions of reward hyposensitivity (215) or hypersensitivity (216). There has also been discussion that energy density, but not sugar specifically plays the most important role in determining the reward value of food (217).

The gateway hypothesis claims that legal drugs (alcohol or nicotine) precedes consumption of cannabinoids, and cannabinoids precede other illicit drugs (218). In animal models of drug abuse this phenomenon appears to be linked to cross-sensitization and instead of increasing locomotor activity it increases the intake of another drug (“consummatory cross-sensitization”) (168). For instance, exposure to cannabis in young adult rats enhanced opiate intake when adults (219). In a separate experiment, pre-exposure of ethanol enhanced cocaine self-administration in adult mice (220, 221). Sugar dependent rats forced to abstain intensified their intake of 9% ethanol. In this case, sugar seems to act as a gateway to alcohol use (222).

Other neurochemical similarities between drugs of abuse and sugar dependent rats have been observed. As previously described in this review, DA response to palatable food habituates following repeated exposure (74, 79), however, when sugar is given intermittently this effect disappears and like drugs of abuse, DA increases every time that the animal is exposed to sugar (79).

Changes in mu-opioid and DA (D1 and D2) properties have also occurred in different experimental models of drug abuse. For instance, repeated cocaine application was correlated with upregulation of mu-opioid receptors (MORs) and increased binding of DA-D1 receptors (223). Self-administration of cocaine in monkeys increased DA-D1 density and decreased DA-D2 receptors (224). However, conflicting results have been detected for the DA-D1 receptor while a consistent DA-D2 receptor downregulation in cocaine addicted subjects occurred (225), alike human studies (112, 226–229). In our sugar intermittent model, an increase in DA-D1 and MOR binding with an opposite response in DA-D2 binding was detected (167). Posteriorly, studies show a decrease in DA-D2 mRNA or binding in the NAc of sugar and high-fructose corn syrup drinkers while the MOR mRNA increased only in high-fructose corn syrup drinkers (230–232). Therefore, palatable food and drugs of abuse share similar neurotransmitter systems with changes in DA release, as well as in receptor function.

In summary, rats in the intermittent sugar access schedule fulfill five of the eleven criteria in the DSM-5 and induces other brain changes that resemble drugs of abuse. Thus, confirming that sugar can be addictive and plays a key role in the broader construct of “food addiction,” at least in this animal model. A brief overview on human data will be summarized below, as well as some of the arguments against FA.

## Addiction potential of highly palatable food related to maternal influence

Given ethical limitations, prospective studies examining the impact of extreme dietary imbalances (high-sugar, or high-fat) during human pregnancy cannot be undertaken. Rodent models show that such dietary extremes (high-sugar and/or high-fat) can impact fetal neurodevelopment, providing evidence of “addiction transfer” from mother to the newborn (233). These animal studies highlight the importance of biological processes (absence of social factors) in the development of FA. Specifically, maternal exposure to drugs of abuse or to highly palatable foods during both the pre- and postnatal period alter behavior via the DA reward system (234, 235) and MOR (236) of the offspring. Intrauterine nutritional experiments in animal models have demonstrated perturbations in hormone (e.g., insulin, leptin, ghrelin) signaling that interact with the development of the reward system in the VTA. Both under- and overfeeding have the potential to increase obesity prevalence in the offspring by way of the DA and opioid systems (237) and such effects have been observed at the intergenerational level (238, 239). Changes in DNA methylation appear to modify genetic expression of DA transporter and MOR (240). While more research has been conducted using a high-fat compared to high-sugar model, caloric sweeteners have been shown to favor hedonic over homeostatic mechanisms (241). Hormonal regulation of food reward may partially explain why sucrose is preferred over artificial sweeteners.

## Human research on “food addiction”

The major construct that has emerged from the theory of FA is the Yale Food Addiction Scale (YFAS). Preliminary validation of the YFAS occurred in 2008 in order to “identify those exhibiting signs of addiction toward certain types of foods” (242). The scale is designed to mirror established alcohol and drug addiction criteria described above. Questions were adapted to assess consumption of high-fat and high-sugar foods and were reviewed by a panel of experts as well as patients with binge eating disorder for feedback on wording. The authors concluded that the YFAS may be a useful tool in identifying individuals with addictive tendencies toward food and propose its use in exploring whether FA is a valid and useful concept. In 2016, the YFAS 2.0 was developed to maintain consistency with the current diagnostic understanding of SUDs described in the DSM-5 which also includes severity indicators (243).

Evidence is accumulating on the overlap of neural circuitry and commonalities between drug abuse and FA in humans (244). Population studies carried out using both YFAS and recently YFAS 2.0 have detected a prevalence of food addicts from as low as 5.4% to as high as 56% depending on the population studied (weighted mean prevalence reported at 19.9% in systematic review) (242, 245–248). Interestingly, this figure [19.9%] closely matches the prevalence of other legal drugs like alcohol (249) and tobacco (250). When considering the association between FA and BMI, close to 20% were obese and little over 40% were underweight (248). One could speculate on the reason of this disparate result. Addictive mechanisms serve a homeostatic function so that if food is scarce one will seek it and binge when found. Additionally, those in the underweight category may be dieting or displaying restrained eating patterns which can increase reward sensitivity for food. The failure of human models of food addiction using YFAS to control for dieting behaviors is a shortcoming of this construct (discussed below).

Dysfunction of the reward system in the presence of highly palatable food becomes a major driver in the prevalence of obesity. While there is an interaction between FA and obesity, they are not the same condition. We cannot discard FA because not all obese people are food-addicted and not all food-addicted are obese (251–253). Many factors are involved in the appearance of obesity and food addiction is just one of them (254), but when 15% of the US population consider themselves as “food addicts” of an estimated 330 million people (census.gov accessed July 2018), then close to 50 million people and (if estimates are correct) close to 20% are obese (248), that gives us a figure of 10 million people that are both food-addicted and obese. This is a substantial number of people with maladaptive functioning. A recent systematic review and meta-analysis of human studies “support that altered general reward-related decision making is a salient neuropsychological factor across eating and weight disorders in adulthood” (255). Taken together, the FA perspective suggests that biochemical changes and genetic predisposition to addiction can lead to excess food consumption independent of social factors. An important theme that has emerged is that FA is both an individual problem as well as a collective problem that should be addressed on a societal level. Given the obesity trends and more recently the opioid epidemic, it can be argued that addiction is the number one public health problem in the United States.

## Food addiction and eating disorders

Research into the interaction between food addiction and eating disorders (EDs), specifically binge eating disorder (BED) and bulimia nervosa (BN), has led to conclusions of separate but related constructs. In one study of individuals with BN, 96% met criteria for FA (244). It has been proposed that those who meet criteria for BN should be separated into distinct subtypes: hyporesponsive to reward (akin to anorexia nervosa) and those with hypersensitive reward circuitry (akin to FA) (256). Approximately half of BED patients meet criteria for FA (257). Overlapping mechanisms include reward dysfunction and impulsivity and unique features for BED include dietary restraint and shape/weight concerns (258).

The biggest gap in our understanding of the interaction between FA and EDs is the restrictive eating component. There are many detractors of the FA hypothesis from the ED treatment community who argue that dieting (also referred to as restrained eating) is what causes elevated scores on the YFAS. It has also been argued that the role played by ingested substances is nonspecific meaning that they apply to EDs as well (259). Future research should control for restrained eating, which has not been adequately done. So, it is not surprising that high prevalence of FA occurs in the underweight category (248, 260) and normal weight category in the case of BN (261). Recently investigators have suggested that FA data can be incorporated into the case conceptualization of EDs from a trans-diagnostic perspective (262, 263). Conclusions suggest giving more consideration to the impact of highly palatable foods for some people seeking ED treatment. A few studies have linked FA and SUD (264, 265) but additional research should be conducted on individuals with SUD in order to further understand how eating behaviors can progress throughout the recovery process. Interaction effects between FA, SUD, and ED have not yet been adequately described.

## Sugar and obesity

Considerable controversy exists with respect to sugar intake and obesity (266). There is general consensus indicating that sugar (sucrose, fructose) is not a direct cause of obesity (267, 268), however, other studies have linked sugar-sweetened beverages (SSB) to an increase in body weight in children and adults (269, 270). Several reasons are offered to explain this discrepancy, but somehow SSB appears to be a special case. First, it is possible that liquid calories are not compensated by a total decrease in energy intake. Second, ingestion of SSB might be an indicator of unhealthy lifestyle (266). None of these studies have linked SSB to sugar addiction so we cannot adequately assess the direct impact of compulsive SSB consumption on body weight.

Nutrition transition theory proposes that “with economic development populations shift from minimally processed diets rich in staple food of vegetable origin to diets high in meat, vegetable oils, and processed foods” (271). As mentioned, this transition in diet is coupled with the obesity epidemic observed in developing countries (272, 273). Research shows that several developing countries in Asia are shifting their diets to preferentially processed foods and carbonated soft-drinks as the main “product vector” for sugar intake (271). Similarly, a shift from minimally processed foods to ultra-processed (more added sugar, more saturated fat, more sodium, less fiber) food has been seen in Brazil (33). Both studies condemned ultra-processed food as an important culprit in the obesity epidemics and ask policy-makers to include legislation and “regulatory approaches” to minimize the impact on health. This approach must be parallel to education programs.

## Policy implications

While ecological approaches targeting global nutrition policy appear promising, agricultural systems remain directed by multibillion-dollar multinational food corporations rather than by governments. It is difficult to predict how emerging data on FA can impact policy, particularly given that corporations have fiduciary responsibilities to their shareholders which require them to maximize profits and may compromise other social and ecological goals (274). Some public health experts propose that we will need to address food corporations similarly to how the tobacco industry was addressed in recent years, with interdiction and litigation (275). It remains unclear how an understanding of FA will translate to behavior change, however, a recent survey suggests that framing certain foods as addictive may increase obesity-related policy support such as warning labels, similar to tobacco (276). Other researchers believe that sugar addiction is too narrow and therefore still premature, warning against policy changes that are unlikely to have an impact since sugar is already so ubiquitous in the food supply (253).

The FA theory directly implicates the food industry, while the nutrition transition theory implicates other global industries also potentially negatively impacting our environment. We propose that the FA framework can lead to improved health outcomes but are more likely to be more pronounced in socially advantaged groups, given barriers created by socioeconomic status. Many public health interventions focused on obesity aim to reduce disparities between groups, which we believe can also have a meaningful impact on long-term health outcomes. Given the evidence reviewed herein, we make the case for sugar addiction in the animal model. Overlooking these findings will represent a missed opportunity for obesity-related policy and a potential public health revolution. Potential treatment strategies for FA have been reviewed elsewhere (277). A commentary on the necessity as well as potential downsides of the food addiction model was published previously (278).

## Conclusion

The FA framework for understanding obesity is the notion that highly processed “hyperpalatable” foods have hijacked the reward centers in the brain thus impairing the decision-making process, similar to drugs of abuse. The major assumption is that biochemistry drives behavior. The sugar addiction theory bridges current gaps between food science and neuroscience, and between nutrition and psychology. This theory was originally developed from animal studies, however there is no shortage of compelling human data. While FA has been sensationalized in the popular press with headlines such as “Oreos More Addictive Than Cocaine?” we propose that processed FA in humans is much more like caffeine or nicotine addiction than it is like cocaine or heroin. There is a subtlety to food addiction where a significant majority of the people who meet criteria may not be aware of it, likely because it is not widely accepted as a social norm. Meanwhile, there have been non-clinical recovery movements of self-identified “food addicts” dating as far back as 1960 when Overeaters Anonymous was formed.

A seminal paper by Glass and McAtee envisioned a future for public health which integrates the natural and behavioral sciences with respect to the study of health. Their multilevel framework extends the “stream of causation” to include both social and biological influences. The authors use the term “embodiment” to describe the “sculpting of internal biological systems that occurs as a result of prolonged exposure to particular environments” (279). These authors propose that next-generation models focus on how social environments affect the organism (human) which will affect the organs, cells, sub-cellular, and molecular levels, and how these will provide feedback at multiple levels. They argue that while social factors act as mediating risk regulators, explanations of obesity must incorporate the biological substrate: “whatever has changed in the environment that has led to exponential expansion in population body weight, must be conspiring with epigenetic and psychophysiological factors. Eating behavior is an example of a phenomenon that results from synergistic interactions among biological (hunger) and social (eating cues) levels” (279).

To date the YFAS is the only validated measure to evaluate addiction-like eating. While there are over 100 original research studies using the YFAS and the tool has undergone several iterations (now YFAS 2.0), brain imaging studies in humans remain somewhat limited, and a gap remains between psychological assessment and reward-related brain circuitry. More importantly, FA research has not been able to account for all of the social factors (e.g., income, education, access, culture) that contribute to food consumption patterns. Additionally, FA is not limited to obesity, as this construct has been extended to non-obese populations which makes causal inference difficult to assess. Much of the appetite-related research does not include the term “food addiction” likely due to the cultural stigmas associated with addiction.

Finally, there is strong evidence of the existence of sugar addiction, both at preclinical and clinical level. Our model has demonstrated that five out of eleven criteria for SUD are met, specifically: use of larger amounts and for longer than intended, craving, hazardous use, tolerance, and withdrawal. From an evolutionary perspective, we must consider addiction as a normal trait that permitted humans to survive primitive conditions when food was scarce. As we evolved culturally, the neural circuits involved in addictive behaviors became dysfunctional and instead of helping us survive they are in fact compromising our health. From a revolutionary perspective, understanding the molecular, and neurological/psychological intricacies of addiction (sugar, drugs of abuse) will permit the discovery of new therapies (pharmacological and non-pharmacological) and possible management of at least one crucial factor in the occurrence of obesity.

## Author contributions

All authors listed have made a substantial, direct and intellectual contribution to the work, and approved it for publication.

### Conflict of interest statement

The authors declare that the research was conducted in the absence of any commercial or financial relationships that could be construed as a potential conflict of interest.
